# Function of *lamp2* Gene Response to *Vibrio vulnificus* Infection and LPS Stimulation in the Half-Smooth Tongue Sole (*Cynoglossus semilaevis*)

**DOI:** 10.3390/ijms26051999

**Published:** 2025-02-25

**Authors:** Tian Han, Yufeng Liu, Mengchao Li, Yitong Zhang, Zhongwei He, Yuqin Ren, Wei Cao, Jiangong Ren, Yufen Wang, Guixing Wang, Chunguang Gong, Jilun Hou

**Affiliations:** 1 Ocean College, Hebei Agricultural University, Qinhuangdao 066009, China; hantian0309@163.com (T.H.); lmc23520@163.com (M.L.); 2 Hebei Key Laboratory of the Bohai Sea Fish Germplasm Resources Conservation and Utilization, Beidaihe Central Experiment Station, Chinese Academy of Fishery Sciences, Qinhuangdao 066100, China; liuyf@bces.ac.cn (Y.L.); yitong_zh@126.com (Y.Z.); hezw@bces.ac.cn (Z.H.); renyq@bces.ac.cn (Y.R.); caow@bces.ac.cn (W.C.); renjg@bces.ac.cn (J.R.); wangyf@bces.ac.cn (Y.W.); wanggx@bces.ac.cn (G.W.); 3 Bohai Sea Fishery Research Center, Chinese Academy of Fishery Sciences, Qinhuangdao 066100, China

**Keywords:** lysosomes, *lamp2*, *Cynoglossus semilaevis*, *Vibrio vulnificus*, LPS

## Abstract

Lysosome-associated membrane glycoproteins (LAMPs), including lysosomal membrane protein 1 (Lamp1) and lysosomal membrane protein 2 (Lamp2), are involved in phagocytosis, chaperone-mediated autophagy (CMA), and other pathways that interact with lysosomal activity. However, the role of Lamp2 in teleosts has not been clarified. In this study, we investigated the functions of *lamp2* genes during *Vibrio vulnificus* infection. We achieved subcellular localization of the *lamp2* gene at the cellular level and performed overexpression and RNA interference experiments followed by Lipopolysaccharides (LPS) stimulation to probe the expression changes of related genes. Ultrapathology analysis of the head-kidney revealed an increase in lysosomes and the formation of autophagosomal vesicles after *V. vulnificus* infection, suggesting that lysosomes bind to autophagosomes. The *lamp2* gene, encoding 401 amino acids in *Cynoglossus semilaevis*, was constitutively expressed in all examined tissues of healthy half-smooth tongue sole, with the highest expression in blood. A challenge test was conducted to assess the response of half-smooth tongue sole (*Cynoglossus semilaevis*) to different concentrations of *V. vulnificus*. The results showed that the relative expression of *lamp2* and its related genes—*lc3*, *rab7*, *vamp8*, *atg14*, *stx17*, *snap29*, *ctsb*, and *ctsd*—varied with time and concentration in the gill, spleen, head-kidney, blood, liver, and gut tissues. From the results of *lamp2* gene overexpression and RNA interference experiments, it is hypothesized that *lamp2* positively regulates *lc3*, *rab7*, *vamp8*, *snap29*, and *stx17*, and negatively regulates *ctsd* and *ctsb*. Our findings provide new primary data for the function of *lamp2* gene in the half-smooth tongue sole., particularly its role in regulating the immune response against *V. vulnificus*.

## 1. Introduction

Lysosomes are organelles found in animal cells that contain enzymes capable of breaking down various biomolecules, including proteins, nucleic acids, and carbohydrates [1]. They play a crucial role in cellular digestion and waste removal. Lysosome-associated membrane glycoproteins (LAMPs), including lysosomal membrane protein 1 (Lamp1) and lysosomal membrane protein 2 (Lamp2), are involved in phagocytosis, chaperone-mediated autophagy (CMA) [2], and other pathways interacting with lysosomal activities. Bacterial infections can affect lysosomes in different ways. For instance, *Mycobacterium tuberculosis* and *Staphylococcus aureus* infections increase the host cellular lysosome levels compared with uninfected conditions, whereas *Salmonella* infection reduces the levels of lysosomal constituents, including *lamp2* [3]. Innate immunity involves the internalization of pathogens, such as bacteria, through phagocytosis and targeting them to lysosomes for degradation [2]. Lysophagic clearance of damaged lysosomes generates lysosomal membrane protein complexes, which constitute small vesicles with the N-terminal protein chain facing the lumen of the vesicle [4]. Overall, lysosomes play a crucial role in degrading cellular waste and pathogens; bacterial infections can affect lysosomal constituents, including *lamp2*.

Recent studies have revealed that Lamp2 exists not only as a structural protein in membrane structures but also has many other functions [5]. For instance, Lamp2 is essential in regulating autoimmune diseases, autophagosome formation, endosomal fusion, cholesterol transport, thymus development, tumor invasion, and metastasis [6]. Lamp2 can be directly involved in neutrophil adhesion, autophagy, and antigen presentation. Several studies have shown that lysosomes and the spread of pathogens are closely linked to many complex biological processes [7].

While more research results are needed to provide a definitive conclusion on the effect of Lamp2 on bacterial infection, some studies have suggested that Lamp2 plays a role in lysosomal function and autophagic flux, which are essential for the degradation of cellular waste and pathogens [4,8]. Additionally, *lamp2* deficiency has been shown to attenuate the neurodegeneration markers induced by HSV-1 infection [9]. Furthermore, Lamp2 has been associated with intracellular bacteria and *Salmonella*-containing vacuoles [10]. Overall, although the exact role of *lamp2* in bacterial infection is unclear, it appears to be involved in lysosomal function and autophagic flux, which are important for pathogen degradation.

The half-smooth tongue sole (*Cynoglossus semilaevis*) is a vital marine fish species in China, particularly significant in aquaculture. However, diseases, especially those caused by bacterial infections, have led to substantial economic losses as the aquaculture industry has expanded [11]. *Vibrio vulnificus*, a zoonotic pathogen, poses a threat to both humans and fish, sometimes leading to sepsis and death. Understanding the interaction between the host and *V. vulnificus* is crucial for insights into the host’s bactericidal activity and the mechanisms of *V. vulnificus* clearance through lysosomal and phagosomal pathways [10,12,13,14,15,16]. In this study, we assessed the ultra-pathological changes associated with *V. vulnificus* infection in the half-smooth tongue sole, characterized the expression of *lamp2*, and analyzed the mRNA expression profiles in various tissues exposed to different concentrations of *V. vulnificus* at distinct time points. Additionally, we profiled the mRNA expression of *lamp2* and its related genes in tissues subjected to Lipopolysaccharides (LPS) stimulation. Our findings offer new insights into the immune function of *lamp2* in the half-smooth tongue sole.

## 2. Results

### 2.1. Sequencing Characterization of lamp2

**Figure 1:**
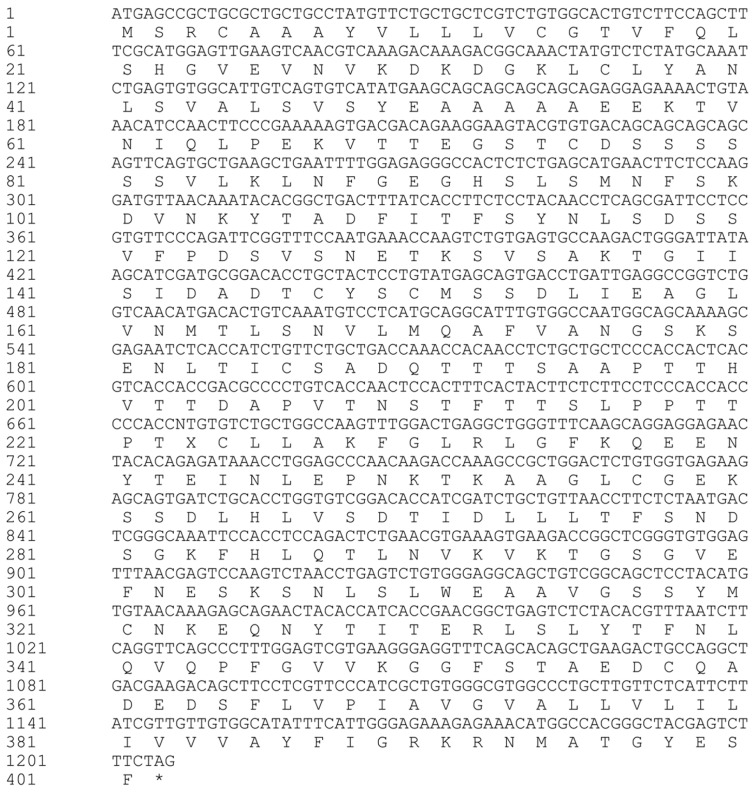
The nucleotide and amino acid sequences of the protein encoded by the CDS region of the *lamp2* gene in *Cynoglossus semilaevis*. (The symbol * denotes the stop codon).

**Figure 2:**
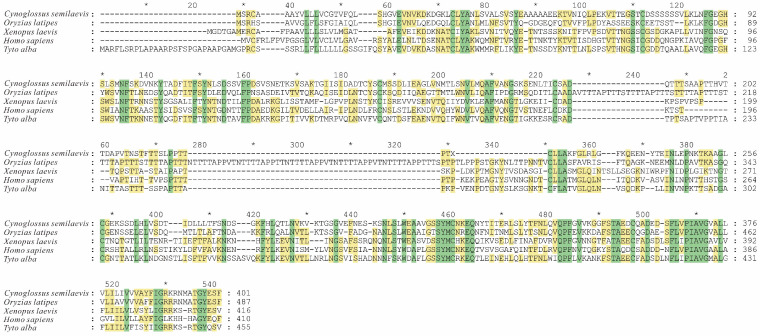
Alignment of multiple Lamp2 amino acid sequences from *Cynoglossus semilaevis* and other species. “*” indicates the position of the amino acid of the sequence, which is used to mark the number of the amino acid in the sequence, green shading indicates amino acid identity; yellow shading indicates similarity (50% threshold).

The open reading frame of the half-smooth tongue sole *lamp2* gene was 1206 bp in length (Figure 1), and encoded 401 amino acids with a predicted molecular weight of 42.91 kDa and a theoretical isoelectric point of 4.84; the probability of *lamp2* having a signal peptide was 98.158%; the signal peptide type was SP (Sec/SPI) secretory signal peptide; the excision site was 23–24; the probability of the presence of a signal peptide was 95.28%; the signal peptide position was 1–23 aa; and the average hydrophobicity was 0.046, which is a hydrophobic protein. The prediction of the transmembrane region revealed that the transmembrane structural domain was located between 365 and 387 amino acids (Figure 2).

**Figure 3:**
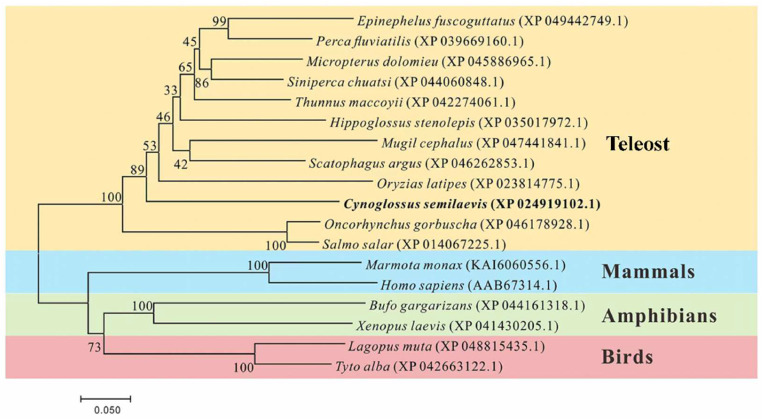
Comparative phylogenetic analysis of the Lamp2 amino acid sequences of *Cynoglossus semilaevis* and other species. The black bold italics font represents the *Cynoglossus semilaevis* branch. (Bootstrap values displayed at nodes).

Phylogenetic analysis revealed that the *lamp2* gene from the half-smooth tongue sole and other teleost fish clustered together, forming a distinct group separate from mammals, amphibians, and birds (Figure 3).

### 2.2. Effect of V. vulnificus on Head Kidney Ultramicroscopic Pathology

**Figure 4:**
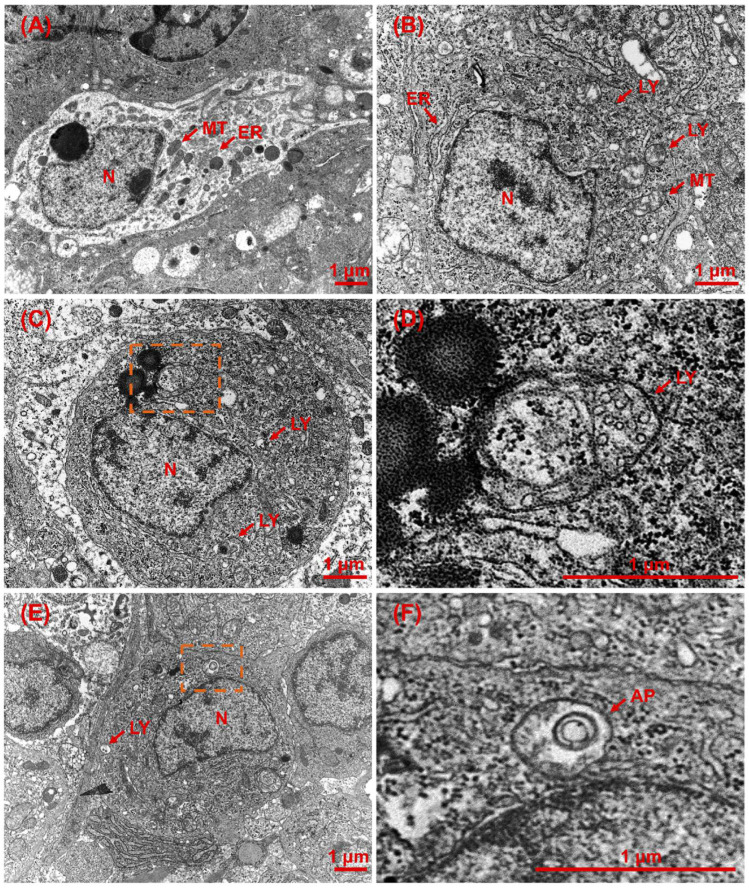
The ultrastructure pathology of tissues in *V. vulnificus* challenge in half-smooth tongue sole (*Cynoglossus semilaevis*) after *V. vulnificus* challenge is depicted as follows: (**A**) shows the head kidney from control group. (**B**,**C**) illustrate the head kidney ultrastructure from 10^11^ CFU/mL group at 72 h post-injection. (**D**) is a magnified view of the area in (**C**), and (**F**) is a magnified view of the area in (**E**). AP, autophagosome; ER, endoplasmic reticulum; N, nucleus; MT, mitochondria; LD, lipid drop; LY, lysosome.

Under ultramicroscopic examination, the head kidney tissue of the healthy half-smooth tongue sole exhibited a uniform cytoplasmic distribution with round or oval nuclei centrally located within the cytoplasm. In these healthy fish, the nuclear membrane boundaries were visible, mitochondria were intact, and the cytoplasm contained numerous neatly arranged endoplasmic reticula (Figure 4A). In contrast, the ultrastructure of the head kidney tissues from infected fish displayed enlarged nuclei with distorted nuclear membrane boundaries. Some nuclei were extruded and displaced from the cell centre; mitochondria appeared swollen and partially lysed; and the endoplasmic reticulum was dilated and disorganized. Furthermore, there was an increase in lysosomes and the formation of autophagic vesicles that bind to lysosomes, as well as the fusion of autophagic vesicles (Figure 4B–F).

### 2.3. Expression of lamp2 in Different Tissues of Half-Smooth Tongue Sole

**Figure 5:**
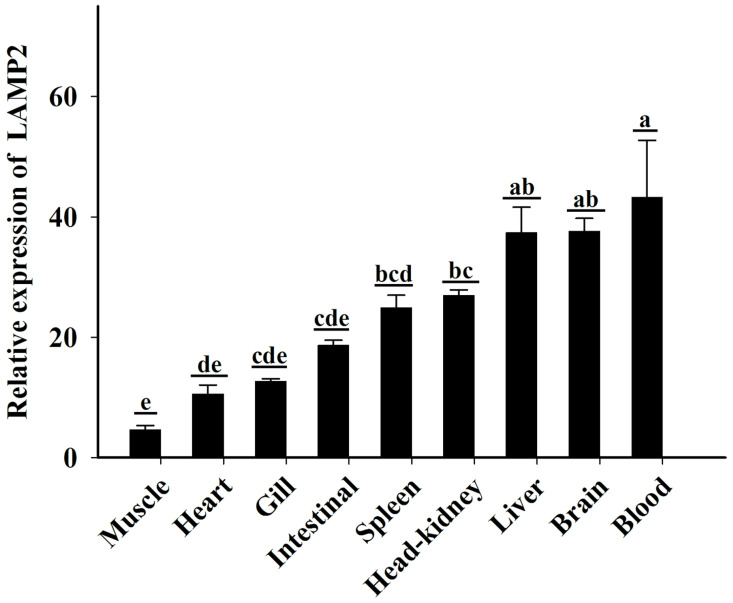
Expression of lamp2 in various tissues of *Cynoglossus semilaevis*. *β-actin* is used as the internal control gene to calibrate the cDNA templates for all the samples. The differential significance of lamp2 expression in different tissues (*p* < 0.05) was expressed by different letters.

The expression of *lamp2* in nine healthy tissues of half-smooth tongue sole was analyzed using qRT-PCR. The results indicated that *lamp2* was expressed in the muscle, heart, gill, intestine, spleen, head kidney, liver, brain, and blood. As can be seen in Figure 5, the relative expression of the *lamp2* gene was highest in blood and lowest in muscle (*p* < 0.01).

### 2.4. Expression of lamp2 in Tissues Stimulated with V. vulnificus

**Figure 6:**
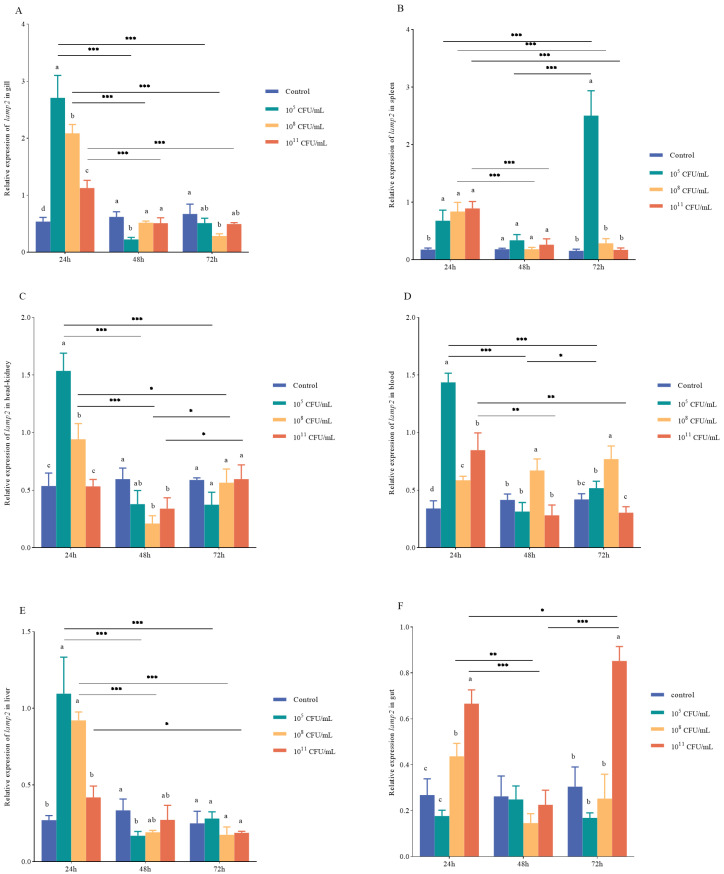
Expression profiles of the *lamp2* gene in various tissues of *Cynoglossus semilaevis* were analyzed following intraperitoneal injection with varying concentrations of *V. vulnificus* at multiple time points. (**A**): gill; (**B**): spleen; (**C**): head kidney; (**D**): blood; (**E**): liver; (**F**): gut. Distinct differences (*p* < 0.05) in *lamp2* expression across varying bacterial concentrations and time points are represented by different letters. Differences in *lamp2* expression at identical bacterial fluid concentrations across various time points are denoted by ‘*’, ‘**’, ‘***’ (‘*’ *p* < 0.05, ‘**’ *p* < 0.01, ‘***’ *p* < 0.001).

Following *V. vulnificus* injection, the differential expression of *lamp2* in gill was observed. At 24 h post-injection, *lamp2* expression was highest in the 10^5^ CFU/mL group, reaching 5.04 times that of the control group. Significant differences in *lamp2* expression were also noted in the 10^8^ and 10^11^ CFU/mL groups compared to the control. At 48 h, significant differences in *lamp2* expression were observed between all experimental groups and the control. At 72 h, a significant difference was found between the 10^8^ CFU/mL and the control. Furthermore, our analysis revealed significant downregulation of *lamp2* expression between 24 and 48 h (*p* < 0.001) and between 24 and 72 h (*p* < 0.001) across all experimental groups (Figure 6A).

The relative expression level of *lamp2* in the spleen following *V. vulnificus* injection demonstrated significant upregulation in the 10^5^ CFU/mL group at 24, 48, and 72 h, with fold changes of 3.86, 1.86, and 16.17, respectively, compared to the control group. At 48 and 72 h, the expression levels in the 10^8^ and 10^11^ CFU/mL groups were not significantly different from the control group (*p* > 0.05). Additionally, the relative expression level in the 10^5^ CFU/mL group was considerably upregulated at 72 h, increasing by 73% compared to the control group (*p* < 0.001). In contrast, the expression of the 10^8^ CFU/mL group was significantly downregulated at both 48 and 72 h (*p* < 0.01), with reductions of 78% at 48 h and 66% at 72 h compared to 24 h (*p* < 0.001). Similarly, the expression in the 10^11^ CFU/mL group was significantly downregulated at both 48 and 72 h, with decreases of 71% at 48 h and 80% at 72 h compared to 24 h (*p* < 0.001) (Figure 6B).

In the head kidney, significant differences in the expression level of *lamp2* were observed among the groups at 24 and 48 h. However, the expression level of *lamp2* was highly upregulated in the 10^5^ and 10^8^ CFU/mL groups at 24 h, with fold changes of 2.86 and 1.75, respectively, compared to the control group. Moreover, at 48 h, the expression levels of the 10^8^ and 10^11^ CFU/mL groups were significantly lower than that of the control group. The expression of *lamp2* was significantly downregulated by 75% at 72 h and 76% at 48 h in the 10^5^ CFU/mL group compared with 24 h (*p* < 0.001) and by 78% at 48 h and 40% at 72 h in the 10^8^ CFU/mL group compared to 24 h (*p* < 0.001; *p* < 0.05). Conversely, in the 10^11^ CFU/mL group, the expression of *lamp2* was significantly upregulated by 12% at 72 h compared with 48 h (*p* < 0.05) (Figure 6C).

The relative expression *lamp2* in the blood following *V. vulnificus* injection indicated high upregulation in the 10^5^ CFU/mL group at 24 h, with levels 4.20 times higher than those in the control group. The expression of *lamp2* across all experimental groups was significantly different from that of the control group. Notably, the 10^8^ CFU/mL group exhibited considerable differential expression of *lamp2* at 48 and 72 h compared to the other groups. Relative to the 24 h result, the expression of *lamp2* in the 10^5^ CFU/mL group was downregulated by 0.78-fold and 0.64-fold at 48 and 72 h, respectively (*p* < 0.001). At 72 h, the expression was significantly higher than at 48 h. In contrast, the 10^11^ CFU/mL group showed a downregulation of *lamp2* by 0.67-fold and 0.64-fold at 48 and 72 h, respectively (*p* < 0.01; *p* < 0.001) (Figure 6D).

In the liver, the relative expression of *lamp2* at 24 h was significantly different in the 10^5^ and 10^8^ CFU/mL groups compared with the control group. At 48 h, the relative expression levels in the 10^5^ CFU/mL group were significantly different from the control group. No significant difference was observed at 72 h. At 48 and 72 h, the expression of *lamp2* was significantly downregulated in the 10^5^ CFU/mL group by 85% and 74%, respectively (*p* < 0.001), compared to 24 h, and in the 10^8^ CFU/mL group by 79% and 81%, respectively (*p* < 0.001), compared with 24 h. However, in the 10^11^ CFU/mL group, the expression of *lamp2* was significantly downregulated by 55% at 72 h compared with 24 h (*p* < 0.01; *p* < 0.05) (Figure 6E).

In the gut, the relative expression of *lamp2* at 24 h was significantly different in the 10^8^ and 10^11^ CFU/mL groups compared to the control group, while no significant difference was observed at 48 h. At 72 h, the relative expression level in the 10^11^ CFU/mL group was significantly different from the other groups. Compared to 24 h, the expression of *lamp2* was markedly reduced in the 10^8^ CFU/mL group at 48 h (*p* < 0.01). In the 10^11^ CFU/mL group, *lamp2* expression was significantly downregulated by 66% at 48 h (*p* < 0.001) but showed an upregulation of 28% at 72 h compared to 24 h (*p* < 0.05). Notably, the expression level was significantly increased by 2.76-fold at 72 h compared with 48 h (*p* < 0.001) (Figure 6F).

### 2.5. Expression of the lamp2-Related Genes in Tissues Stimulated with V. vulnificus

**Figure 7:**
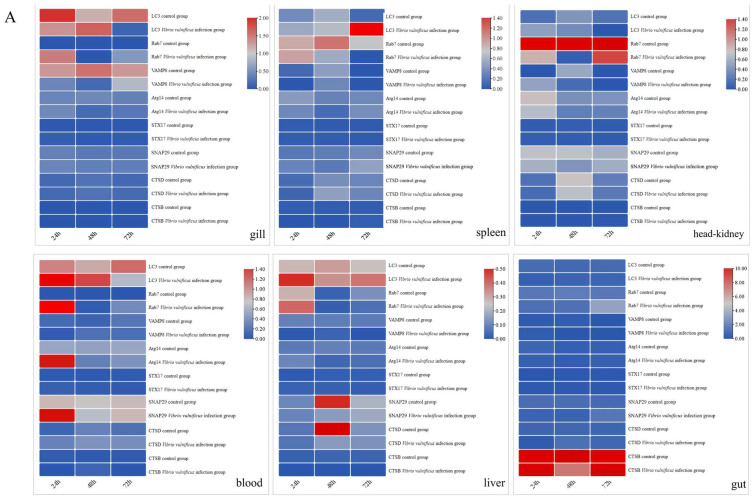

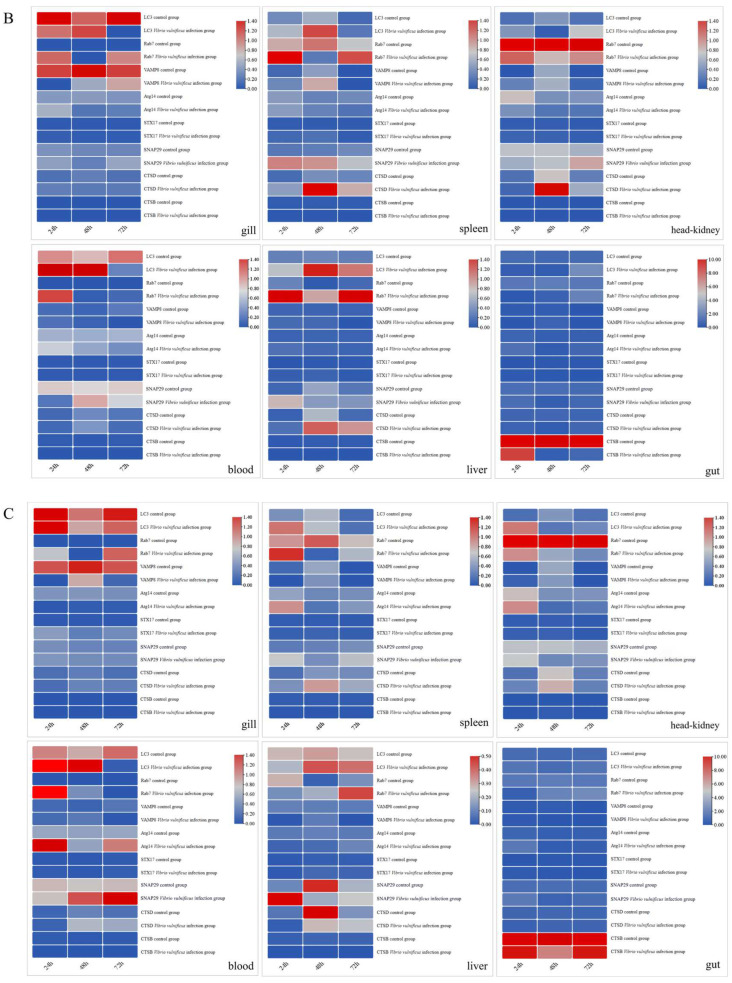
Temporal expression of *lamp2*-related genes is detected in gill, spleen, head kidney, blood, liver, and gut by qRT-PCR after different concentrations of *V. vulnificus* infection (24 h, 48 h, 72 h). (**A**): The *V. vulnificus* concentration is 10^5^ CFU/mL; (**B**): the *V. vulnificus* concentration is 10^8^ CFU/mL; (**C**): the *V. vulnificus* concentration is 10^11^ CFU/mL; *β-actin* is used as the internal control gene to calibrate the cDNA templates for all the samples. The heatmap is constructed by TBtools software (v2.142).

No notable distinction in the expression of *lc3* was observed in the gills among all experimental groups injected with *V. vulnificus* at 24 and 48 h. In comparison, at 72 h, the expression of *lc3* was markedly reduced in the 10^5^ CFU/mL group (*p* < 0.01) and the 10^8^ CFU/mL group (*p* < 0.05). Conversely, the expression of *lc3* was significantly upregulated in the 10^11^ CFU/mL group at 72 h compared with the 10^5^ and 10^8^ CFU/mL groups (*p* < 0.05). The expression of *rab7* was significantly upregulated in the 10^8^ CFU/mL group at 24 h (*p* < 0.05) and in the 10^11^ CFU/mL group at 72 h (*p* < 0.05). The expression of *vamp8* was drastically decreased in the 10^11^ CFU/mL group at 24 h (*p* < 0.05), and it was significantly downregulated in the 10^8^ CFU/mL group at 24 h (*p* < 0.05). No significant differences were observed in the expression of *atg14*, *stx17*, *snap29*, *ctsd*, and *ctsb* genes (*p* ≥ 0.05) (Figure 7A–C).

In the spleen of the 10^5^ CFU/mL group, the expression of *lc3* was significantly upregulated at 72 h compared with the control group and increased significantly at 72 h compared to 24 and 48 h (*p* < 0.01). However, in the 10^8^ CFU/mL group, the expression of *lc3* was markedly increased at 48 h compared to 72 h and the control group (*p* < 0.01). In the 10^11^ CFU/mL group, *lc3* expression was drastically decreased at 72 h compared to 24 h (*p* < 0.05) and significantly upregulated at 48 h (*p* < 0.05). In the 10^8^ CFU/mL group, the *lc3* was substantially raised at 48 h (*p* < 0.05) and markedly reduced at 72 h in contrast with 24 h (*p* < 0.05). The expression of *rab7* in the 10^8^ CFU/mL group was significantly downregulated (*p* < 0.05) at 48 h, while in the same group, it was substantially raised at 24 h and downregulated at 72 h. In the 10^11^ CFU/mL group, *rab7* expression was drastically decreased at 48 h compared with the control group. The *snap29* expression was substantially elevated in the 10^11^ CFU/mL group at 24 h in contrast with the control group. In the 10^11^ CFU/mL group, the expression of *ctsd* was significantly decreased at 48 h (*p* < 0.01), while there were no significant differences in the expressions of *atg14*, *stx17*, and *ctsb* (*p* ≥ 0.05) (Figure 7A–C).

In the head kidney, the expression of *rab7* was markedly reduced in the 10^5^ CFU/mL group at 24 h (*p* < 0.05) and 48 h (*p* < 0.01) but significantly upregulated at 72 h compared with 24 and 48 h (*p* < 0.05). In the 10^8^ CFU/mL group, *rab7* expression was significantly elevated at 48 and 72 h (*p* < 0.05). In the 10^11^ CFU /mL group, *rab7* expression was significantly decreased at 72 h (*p* < 0.05). Meanwhile, *ctsd* expression was significantly upregulated at 48 h (*p* < 0.01) and markedly increased at 48 h compared with 24 h (*p* < 0.001). In all experimental groups, there were no significant differences in the expression of the *lc3*, *vamp8*, *atg14*, *stx17*, *snap29*, and *ctsb* genes (*p* ≥ 0.05) (Figure 7A–C).

In the blood, the expression of *lc3* in the 10^5^ CFU/mL group was markedly increased at 24 h compared to the control group (*p* < 0.05). However, at 72 h, *lc3* expression was significantly downregulated compared with 24 h (*p* < 0.01). Additionally, *rab7*, *atg14*, and *snap29* were markedly increased at 24 h compared with the control group (*p* < 0.01) but were markedly reduced at 48 and 72 h relative to 24 h (*p* < 0.01). In the 10^8^ CFU/mL group, *lc3* expression was significantly elevated at 24 and 48 h compared with the control group (*p* < 0.01), but was markedly reduced at 72 h compared to 24 and 48 h (*p* < 0.05). In the 10^8^ and 10^11^ CFU/mL groups, *rab7* was greatly elevated at 24 h compared to the control group and was significantly downregulated at 48 and 72 h compared with 24 h (*p* < 0.001). In the 10^11^ CFU/mL group, *lc3* expression was significantly increased at 24 and 48 h (*p* < 0.05) and substantially reduced at 72 h compared with 24 and 48 h (*p* < 0.001), atg14 expression was significantly upregulated at 24 h, and *snap29* expression was markedly increased at 48 and 72 h compared to the control group (*p* < 0.05) and was consistently upregulated at 48 h and 72 h compared to 24 h (*p* < 0.01). In contrast, the expressions of *vamp8*, *stx17*, *ctsd*, and *ctsb* were not significantly different (*p* ≥ 0.05) (Figure 7A–C).

In the liver of the 10^5^ CFU/mL group, the expression of *lc3* was significantly upregulated at 24 h in contrast with the control group (*p* < 0.05). The expression of *rab7* was drastically decreased at 48 and 72 h compared with 24 h (*p* < 0.05). S*nap29* and *ctsd* were considerably downregulated at 48 h (*p* < 0.01). In the 10^8^ CFU/mL group, the expression of *lc3* was markedly increased at 48 h compared to the control group (*p* < 0.05), and *rab7* expression was significantly upregulated at 24 h and 72 h (*p* < 0.05). In the 10^11^ CFU/mL group, the expression of *lc3* was substantially raised at 48 h compared with the control (*p* < 0.05), *rab7* expression was markedly increased at 72 h (*p* < 0.001), and Snap29 expression was greatly increased at 24 h but was significantly downregulated at 48 h. *Ctsd* expression was also significantly downregulated at 48 h. The expressions of *vamp8*, *atg14*, *stx17*, and *ctsb* were not significantly different (Figure 7A–C).

In the gut, *ctsb* expression was greatly decreased at 48 and 72 h in the 10^8^ CFU/mL group compared with the control group and was drastically lowered at 48 and 72 h compared with 24 h. No significant differences were observed for the expressions of *lc3*, *rab7*, *vamp8*, *atg14*, *stx17*, *snap29*, and *ctsd* (Figure 7A–C).

### 2.6. Subcellular Localization of lamp2 Gene

**Figure 8:**
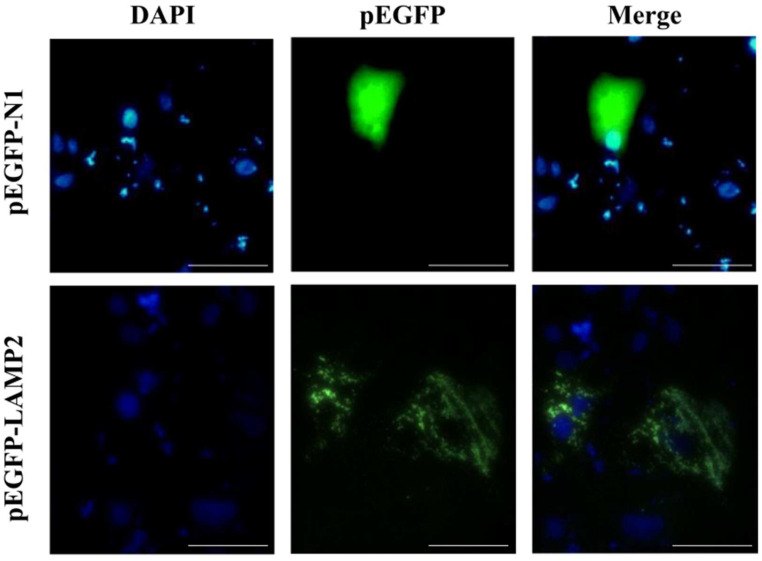
Localization of Lamp2 within the cytoplasm of *Cynoglossus semilaevis* brain cells (CSBCs). CSBCs were transfected with pEGFP-N1, pEGFP-LAMP2, and nuclei were stained with DAPI. Observe the cells under a fluorescence microscope (the scale’s length is 50 μm).

To determine the subcellular localization of *lamp2* in vitro, pEGFP-N1 and pEGFP-*lamp2* plasmids were transfected into *Cynoglossus semilaevis* brain cells (CSBCs) and the nuclei were counterstained with DAPI. As shown in Figure 8, green fluorescence was observed in the cytoplasm and nucleus of cells transfected with pEGFP-N1. In contrast, green fluorescence was detected exclusively in cells transfected with pEGFP-*lamp2* cytoplasm. These results suggest that *lamp2* is a cytoplasmic protein.

### 2.7. Expression of Relevant Genes in LPS-Stimulated Cells After lamp2 Overexpression and RNAi

#### 2.7.1. Expression of Related Genes After *lamp2* RNAi 

**Figure 9:**
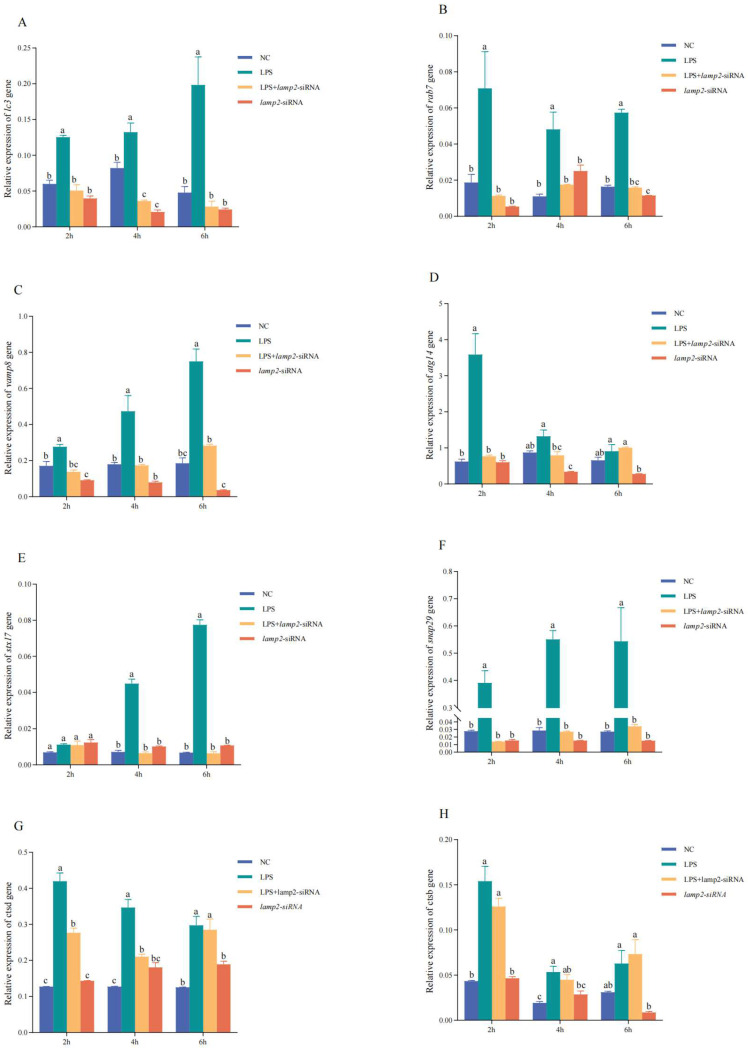
Investigations related to RNA interference. To determine the biological role of *lamp2* in CSBCs, RT-qPCR was employed to assess the impact of *lamp2* gene interference on the expression levels of *lc3*, *rab7*, *vamp8*, *atg14*, *snap29*, *stx17*, *ctsd*, and *ctsb* at various time points (2, 4, 6 h). (**A**): Gene expression of *lc3*. (**B**): Gene expression of *rab7*. (**C**): Gene expression of *vamp8*. (**D**): Gene expression of *atg14*. (**E**): Gene expression of *stx17*. (**F**): Gene expression of *snap29*. (**G**): Gene expression of *ctsd*. (**H**): Gene expression of *ctsb*. The significance of the differences in gene expression (*p* < 0.05) between different groups and time points was expressed in different letters.

The RNAi experiments yielded intriguing results regarding the expression patterns of autophagy-related genes in CSBCs across four distinct experimental groups (NC, LPS, LPS + *lamp2*-siRNA, and *lamp2*-siRNA) at 2, 4, and 6 h (Figure 9A–H). RT-qPCR analysis reveals that the expression levels of *lc3*, *rab7*, *vamp8*, *atg14*, *snap29*, *stx17*, *ctsd*, and *ctsb* were significantly upregulated at various times points in the LPS group compared to the NC group (*p* < 0.001). Conversely, in the *lamp2*-siRNA group, the expression of *lc3*, *rab7*, *vamp8*, *atg14*, and *ctsd* was significantly downregulated (*p* < 0.05). Furthermore, the gene expression levels of *lc3*, *rab7*, *vamp8*, *snap29*, and *stx17* were notably downregulated (*p* < 0.05) at the 4 and 6 h in the LPS + *lamp2*-siRNA group relative to the LPS group. In contrast, *atg14* and *ctsd* were significantly downregulated only at 4 h (*p* < 0.05). These findings underscore the dynamic interplay between LPS stimulation and *lamp2* gene silencing in modulating autophagy-related gene expression.

#### 2.7.2. Expression of Related Genes After *lamp2* Overexpression

**Figure 10:**
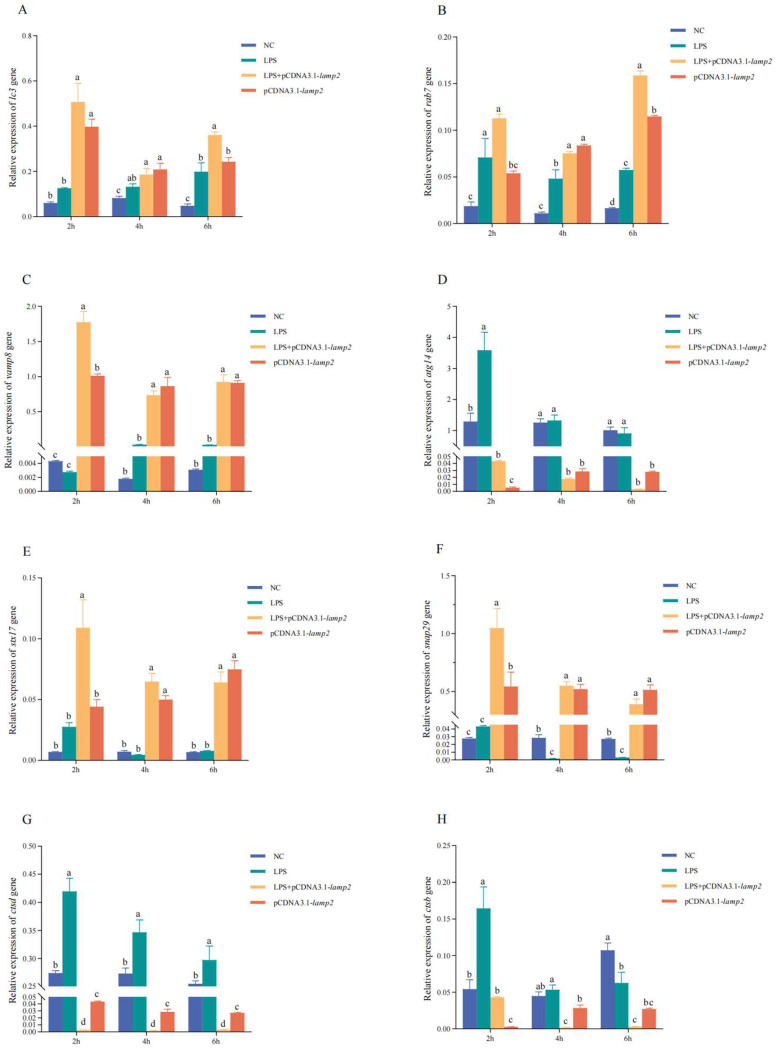
Overexpression experiments. To explore the biological function of *lamp2* in CSBCs, influences of *lamp2* on the expression of *lc3*, *rab7*, *vamp8*, *atg14*, *snap29*, *stx17*, *ctsd*, and *ctsb* were detected by RT-qPCR at different times (2, 4, 6 h). (**A**): Gene expression of *lc3*. (**B**): Gene expression of *rab7*. (**C**): Gene expression of *vamp8*. (**D**): Gene expression of *atg14*. (**E**): Gene expression of *stx17*. (**F**): Gene expression of *snap29*. (**G**): Gene expression of *ctsd*. (**H**): Gene expression of *ctsb*. The significance of the differences in gene expression (*p* < 0.05) between different groups and time points was expressed in different letters.

To investigate the impact of *lamp2* on the expression of autophagy-related genes, including *lc3*, *rab7*, *vamp8*, *atg14*, *snap29*, *stx17*, *ctsd*, and *ctsb*. RT-qPCR analysis was conducted on samples from four distinct groups, as depicted in Figure 10A–D. Following transfection of CSBCs with the pcDNA3.1*-lamp2* plasmid, a significant upregulation in the expression levels of *lc3*, *rab7*, *vamp8*, *snap29*, and *stx17* genes was observed in the pcDNA3.1-*lamp2* group compared to the NC group (*p* < 0.01). In contrast, the expression of the *atg14*, *ctsd*, and *ctsb* genes was significantly downregulated (*p* < 0.01). In the LPS + pcDNA3.1-*lamp2* group, the combination of LPS and pcDNA3.1-*lamp2* resulted in a synergistic and significant increase in the expression of *vamp8*, *snap29*, and *stx17* at 2, 4, and 6 h, *lc3* at 2 h and 6 h, and *rab7* at 4 h and 6 h. These increases surpassed the levels observed in both the NC and LPS groups (*p* < 0.05). Conversely, the expression of the *atg14*, *ctsd*, and *ctsb* genes was markedly downregulated under these conditions (*p* < 0.05). These findings underscore the complex regulatory role of *lamp2* in modulating the expression of key autophagy genes in response to LPS stimulation.

## 3. Discussion

The expression of *lamp2* in the inflamed human liver was associated with the body’s immune state [17]. In the current study, the expression of *lamp2* was significantly upregulated after *V. vulnificus* infection, and the expression of its upstream and downstream genes also changed significantly. In the context of *V. vulnificus* infection, Rab7 is a protein that plays a crucial role in the endocytic pathway and is responsible for the uptake and processing of various molecules in cells. It is involved in transporting vesicles from early endosomes to late endosomes and lysosomes; studies have shown that the bacterium can manipulate the host cell’s endocytic pathway by interacting with Rab7 [18]. This interaction allows *V. vulnificus* to evade the host’s immune response and establish a successful infection. *V. vulnificus* infection of fish causes autophagy in the form of macroautophagy [19,20], which is mediated by double-membrane autophagosomes that enclose cytosolic cargoes, followed by fusion with late endosomes/lysosomes for degradation [21,22]. Stx17 localized to autophagosomes is essential for autophagosome–lysosome fusion as it interacts with Snap29 and Vamp8 localized to late endosomes/lysosomes. Atg14 localized to autophagosomes enhances autophagic fusion by interacting with the Stx17–Snap29 complex [23]. The upregulated expression of these genes indicates an increase in the number of lysosomes, which is consistent with the results of TEM. It can be hypothesized that the rise in the number of lysosomes in the cells of different tissues after *V. vulnificus* infection caused cellular autophagy and fused with intracellular autophagosomes to form autophagic lysosomes, which are digested and hydrolyzed by hydrolases in the lysosomes.

In our study, stimulation of CSBCs with LPS led to a marked increase in the expression of key autophagy-related genes, including *lc3*, *rab7*, *vamp8*, *atg14*, *snap29*, *stx17*, *ctsd*, and *ctsb*. In a transgenic rainbow trout model, *lamp2* gene expression was significantly upregulated in the lysosomal/phagocytic pathway following the tapping assay [24]. Interestingly, treatment with LPS and *lamp2*-siRNA resulted in a significant downregulation of *lc3*, *rab7*, *vamp8*, *atg14*, *snap29*, and *stx17*, while *ctsd* and *ctsb* expression levels were notably upregulated. Conversely, LPS stimulation after *lamp2* overexpression showed an upregulation of *lc3*, *rab7*, *vamp8*, *atg14*, *snap29*, and *stx17*, alongside a significant downregulation of *ctsd* and *ctsb*. These data indicate that *lamp2* has a regulatory function in the expression among these genes. *Lamp2* appears to act through the phosphorylation of *vamp8* by mTORC1, which negatively regulates autophagy, particularly during the maturation phase of autophagosomes [25]. Vamp8, an R-SNARE protein localized in lysosomes, governs the fusion of autophagosomes with lysosomes. Under autophagy-inducing conditions, mTORC1-mediated phosphorylation of Vamp8 is significantly reduced, while the dephosphorylated form of vamp8 robustly promotes the formation of the Stx17–Snap29–Vamp8 complex [25]. Stx17, an autophagosome-localized Qa protein, along with Snap29 and Vamp8, facilitates the fusion of autophagosomes with lysosomes [26]. The acetylation of Stx17, controlled by HDAC2, influences autophagosome maturation. Furthermore, Stx17 promotes translocation from the Golgi to the pre-autophagic structure (mPAS) through TBK1 phosphorylation [27]. Snap29, a Qbc protein, together with Stx17 and Vamp8, forms a SNARE complex that enhances autophagosome–lysosome fusion, and its O-GlcNAcylation modulates interaction with Stx17 [28]. In a zebrafish model, the assembly and function of the Stx17–Snap29–Samp8 complex were closely associated with autophagy. The RUNDC1 protein was identified as an inhibitory regulator of this complex, impacting the fusion of autophagosomes with lysosomes and the fish’s response to nutrient deficiencies and pathogen infections [29]. The Stx17–Snap29–Vamp8 complex also plays a crucial role in fish immune responses, with bacterial infection activating the STING pathway, which regulates the assembly of Stx17 with the Snap29–Vamp8 complex, thereby affecting autophagic flux and immune responses. This mechanism may be pivotal in fish resistance to bacterial and viral infections [26,30,31]. A synergistic role between Lamp2 and the Stx17–Snap29–Vamp8 complex in autophagy has been proposed, with Lamp2, as a lysosomal membrane protein, being involved in the recognition and fusion process between autophagosomes and lysosomes along with the Stx17–Snap29–Vamp8 complex [32]. Given the significance of autophagy in the cellular clearance of pathogens and maintenance of cellular homeostasis, regulating the Stx17–Snap29–Vamp8 complex may offer new targets for controlling bacterial and viral diseases in fish.

During autophagy, Lamp2 regulates the immune response and both macroautophagy (MA) and molecular chaperone-mediated autophagy (CMA) [33]. As major lysosomal proteases, Ctsd and Ctsb are involved in protein degradation, and Lamp2 may indirectly regulate their activities by affecting lysosomal function [34,35,36]. Under steady-state conditions, Ctsb can cleave the calcium channel Mcoln1/Trpml1 in lysosomes, maintaining the inhibition of the transcription factor TFEB and reducing the expression of lysosomal and autophagy-associated proteins, thus controlling lysosome and autophagosome numbers. Since TFEB is a key transcription factor for lysosomal biogenesis and autophagy, Ctsb, by affecting TFEB activity, may subsequently influence Lamp2 expression and function [35]. Newcastle disease virus (NDV) utilizes the sialidase activity of its HN proteins to hydrolyze sialic acid residues on Lamp1 and Lamp2 glycan chains, leading to deglycosylation and degradation of Lamp1 and Lamp2 by Ctsb within the lysosomal lumen, triggering lysosomal membrane permeability (LMP) and apoptosis. This discovery reveals a direct role for Lamp2 in regulating Ctsd and Ctsb activity by preventing their aberrant release by maintaining lysosomal membrane integrity [37].

Therefore, we hypothesized that the *lamp2* gene has earlier and higher expression in the gill, blood, kidney, and liver. In this experiment, we used *V. vulnificus* for intraperitoneal injection of fish, which causes an autophagic response, thus affecting the expression differences in the *lamp2* gene and its upstream and downstream genes in the autophagic pathway. The results of *lamp2* gene overexpression and RNA interference experiments hypothesized that *lamp2* positively regulated *lc3*, *rab7*, *vamp8*, *snap29*, and *stx17* and negatively regulated *ctsd* and *ctsb*.

## 4. Materials and Methods

### 4.1. Experimental Fish and Vibrio vulnificus

The half-smooth tongue sole fish were obtained from Tangshan Sheng Ru Aquaculture Company (Tangshan, Hebei, China). The fish were 67.0 ± 10.0 g in body weight and 22.0 ± 3.0 cm in length. Before the experiment, the fish were cultured in aquariums for 2 weeks. The water temperature before and during the experiment was maintained at 23–25 °C. A total of 360 fish were selected for this study, divided into three treatments groups and one control group, with three parallel fish in each group and 30 fish in each parallel group.

*V. vulnificus* (ATCC 27562) [38] was obtained from the Marine Culture Collection of China (Xiamen, Fujian, China). The culture of *V. vulnificus* was performed according to the previously described method [11,39].

### 4.2. V. vulnificus Injection

The concentrations of *V. vulnificus* injection were set as 1 × 10^5^ CFU/mL, 1 × 10^8^ CFU/mL, and 1 × 10^11^ CFU/mL according to the protocol of Liu et al. [11].

The control group was injected with 100 μL of 1× PBS. At 24, 48, and 72 h after injection, samples of the gill, spleen, head kidney, blood, liver, and gut were collected from each group and quickly frozen in liquid nitrogen for 48 h, then stored at −80 °C.

### 4.3. Sequence Clone and Analysis

Using the half-smooth tongue sole cDNA sequence sample, we cloned the CDS region of *lamp2* using the conventional PCR method [40,41] and head kidney transcriptome. The primers utilized in this study are detailed in Table 1. Homology analysis of *lamp2* was conducted using the BLAST program (2.13.0). The ExPASy translate tool (https://web.expasy.org/translate/ (accessed on 20 July 2022) was employed for amino acid sequence analysis, and molecular weights were determined using the Compute pI/Mw software (http://web.expasy.org/protparam/ (accessed on 20 July 2022). Protein structure prediction was performed using the NCBI Conserved Domain program (https://www.ncbi.nlm.nih.gov/Structure/cdd/cdd.shtml (accessed on 22 July 2022). Multiple sequence alignment was carried out using the ClustalX1.83 software, and the data were edited using the END script program. The phylogenetic tree (bootstrap 1000) was constructed using the Neighbor-Joining (NJ) method in the MEGA X software [42].

### 4.4. qRT-PCR

To understand the expression profile of *lamp2* and its associated genes in the experiment, total RNA was extracted using Trizol reagent (Invitrogen, Carlsbad, CA, USA) following the manufacturer’s instructions. The RNA concentration was determined using a microspectrophotometer (Implen, Munich, Germany), and RNA quality was assessed by nucleic acid electrophoresis. The volume required for reverse transcription of 1 µg of RNA was calculated based on the measured RNA concentration. cDNA was synthesised using the PrimeScript™ RT reagent kit with gDNA Eraser (Perfect Real Time) (TAKARA, Kyoto, Japan), and subsequently diluted 5-fold with DEPC water. This was followed by qRT-PCR analysis conducted on the Novogene q225 platform, with primers designed using Primer 5 software at a concentration of 10 µM. Each time point included three replicates. The qRT-PCR reaction system and conditions were based on the study by Liu et al. [11], and the relative expression of the genes was evaluated using the 2^−ΔΔCt^ method [43].

### 4.5. Transmission Electron Microscopy

The TEM experimental method was adapted from the study of Liu et al. [11]. Head kidney samples from the 10^11^ CFU/mL and PBS groups were fixed overnight in an electron microscopy fixative solution (Servicebio, Wuhan, China) and protected from light exposure. Subsequently, the samples were sent to Servicebio Biotech (Wuhan, China) for observation under the transmission electron microscope.

### 4.6. Subcellular Localization

In reference to the research by Guo et al. [44], CSBCs were cultured in 12-well plates until they reached 70–90% confluence for subcellular localization studies. Plasmid–lipid complexes were prepared according to the protocol provided by GeticoFect 3000 Transfection Reagent (Genetic Biotech, Wuxi, China). Specifically, the plasmids pEGFP-N1 and pEGFP-*lamp2*, along with the Lipo3000 reagents, were each diluted with Opti-MEM serum-free medium (Gibco, Carlsbad, CA, USA). The two diluents were then combined and incubated at room temperature for 15 min. The mixture was distributed evenly among the cells, which were incubated at 23 °C for 4 h. After this incubation period, the medium was replaced, and the cells were further incubated for 24 h. Following this, the medium was discarded, and the cells were washed with PBS and fixed with 4% PFA for 30 min. The cells were then washed twice with PBS for 10 min each. Nuclei were stained with DAPI for 10 min and rinsed with PBS in the dark five times. Finally, the cells were examined using a fluorescent microscope (Leica, Vizla, Germany).

### 4.7. Cell Treatments

The medium was changed on the first day of cell culture, and CSBCs were subjected to LPS challenge. Referring to the research of Cheng et al. [45], the experiment consisted of seven groups, each with six replicates: negative control (NC); LPS; *lamp2*-small interfering RNA (siRNA); *lamp2*-siRNA + LPS; pcDNA3.1-*lamp2* plasmid; and LPS + pcDNA3.1-*lamp2*. RNA extraction was performed at 2, 4, and 6 h post-treatment.

#### 4.7.1. RNA Interference (RNAi) Experiments

The *lamp2*-siRNA interfering strand was synthesized by GENCEFE Biotech (Wuxi, China). The sequences of the interfering strands, designated as *lamp2*-siRNA-F and *lamp2*-siRNA-R, are presented in Table 1. Following the protocol provided by the GeticoFect 3000 Transfection Kit (Genetic Biotech, Shanghai, China), the *lamp2*-siRNA and control siRNA reagents were introduced into CSBCs as detailed below: In tube A, 125 μL of Opti-MEM serum-free medium (Gibco, Carlsbad, CA, USA) was mixed with 5 μL of siRNA. In tube B, 125 μL of Opti-MEM serum-free medium was combined with 7.5 μL of Lipo3000 and incubated at room temperature for 15 min. The contents of tube B were then added dropwise to tube A. After 4 h of incubation, the medium was replaced with a complete medium, and the cells were further incubated at 23 °C for 24 h. Subsequently, LPS was added at a concentration of 100 μg/mL to stimulate the cellular response.

#### 4.7.2. Overexpression Experiments

The pcDNA3.1-*lamp2* plasmid was synthesized by GENCEFE Biotech. The method described in Section 4.6 for the subcellular localization experiments was followed for the transfection of cell overexpression plasmid. After 4 h, the medium was changed to a complete medium, and 24 h later, LPS (100 μg/mL) was added to stimulate the cellular response. For subsequent studies, eight genes related to the expression of *lamp2* were selected: *lc3*, *rab7*, *vamp8*, *atg14*, *snap29*, *stx17*, *ctsd*, and *ctsb*. The primer information for these genes is detailed in Table 1.

### 4.8. Statistical Analysis

We conducted a Shapiro–Wilk test to evaluate the normality of the data distribution, and the results confirmed that the data were normally distributed. Given these findings, we employed one-way ANOVA followed by Tukey’s post hoc test (*p* < 0.05) to compare the qRT-PCR data. The data are presented as the mean ± standard error of the mean (SEM). For statistical analyses, we utilized R software (version 4.4.0) with the stats package (4.4.0) [46].

## 5. Conclusions

The present study characterized *lamp2* in teleost for the first time and investigated its function in response to *V. vulnificus* infection. Injection *V. vulnificus* caused a significant upregulation of *lamp2* expression and an increase in the number of phagosomes and lysosomes. Our findings indicate that the abnormal expression of *lamp2* in *V. vulnificus*-injected half-smooth tongue sole may be related to the immune response evoked by *V. vulnificus,* which affects the differential expression of autophagosome and lysosome fusion-associated proteins.

## Figures and Tables

**Table 1:** Primer sequence table.

Primers	Sequence (5′-3′)	Applications
pEGFP-LAMP2-F	AGCTCAAGCTTCGAATTCATGAGCCGCTGCGCTGCTGCCTATG	Eukaryotic expression
pEGFP-LAMP2-R	GTCGACTGCAGAATTCCTAGAAAGACTCGTAGCCCGTGGCC	
Q-*lamp2*-F	ACCGCATCCAACGCCAAAACC	qRT-PCR
Q-*lamp2*-R	CGCTTGCTGGGCTGGTGAAGA	
β-actin-F	AGGGAAATCGTGCGTGACAT	
β-actin-R	GCCCATCTCCTGCTCGAA	
CS-*lc3*-F	ACCAGCATAGTATGGTGTCAGTG	
CS-*lc3*-R	AAAGTCTCCTGGGAGGCGTA	
CS-*rab7*-F	CCAGTACAAAGCCACAATAGG	
CS-*rab7*-R	TCCACGGTAGAACGCAACAC	
CS-*stx17*-F	CGCTGATGCCCTGGAAATGC	
CS-*stx17*-R	GGAGGTGATGGCGGTGGTGT	
CS-*snap29*-F	TGCGACAGGGTGAGGTTCTG	
CS-*snap29*-R	ACACTCTTGATGCTGCTAATG	
CS-*vamp8*-F	GGTGGTGGTCATTATTGTGG	
CS-*vamp8*-R	GTTTATGGCTTCTTGGTGGG	
CS-*ctsd*-F	TGCCGTCCATTCACTGCTCC	
CS-*ctsd*-R	CGAAGGCTGTGCCGTTCTTG	
CS-*ctsb*-F	TGTCAGGGTGAACAGGAAAC	
CS-*ctsb*-R	GACGGGACGCTGTAGATTTT	
CS-*atg14*-F	GAAGAGCACCAACCAGGGTC	
CS-*atg14*-R	CCAGGATGTGGGAAAGAATGT	
*lamp2*-siRNA-F	CAUCUGUUCUGCUGACCAATT	RNA interference
*lamp2*-siRNA-R	UUGGUCAGCAGAACAGAUGTT	

Note: the primer concentration is 10 µM.

## Data Availability

Data are contained within the article.
